# Ventricular and lumbar cerebrospinal fluid analysis in 77 HIV-negative patients with *Cryptococcal* meningitis who received a ventriculoperitoneal shunt

**DOI:** 10.1038/s41598-022-25742-w

**Published:** 2022-12-09

**Authors:** Qing Dong, Zhenchao Huang, Peng Yu, Enpeng Song, Zhijie Chen, Feng Qin

**Affiliations:** 1 Department of Neurology, The Third Affiliated Hospital of Sun Yat-Sen University, No. 600 Tianhe Road, Guangzhou, 510630 People’s Republic of China; 2 Department of Neurosurgery, Lingnan Hospital, Branch of the Third Affiliated Hospital of Sun Yat-Sen University, No. 2693 Kaichuang Avenue, Guangzhou, 510530 People’s Republic of China

**Keywords:** Anatomy, Diseases, Medical research, Neurology

## Abstract

Lumbar cerebrospinal fluid (CSF) parameters are widely studied and have wide clinical applications, but ventricular CSF has rarely been studied since it is relatively difficult to obtain. To determine whether there are differences between ventricular and lumbar CSF parameters and whether the differences have clinical significance, we retrospectively reviewed 77 patients with *Cryptococcal* meningitis who received a ventriculoperitoneal shunt. We analyzed the following parameters: white blood cell count, total protein concentration, CSF/blood glucose ratio, chloride ion concentration, and *Cryptococcal* count. All parameters between lumbar and ventricular CSF were remarkably different (all *p* < 0.001). White blood cell count, total protein level and *Cryptococcal* count were lower in ventricular CSF than in lumbar CSF, while CSF/blood glucose ratio and chloride ion concentration were higher. Compared to patients without ventriculomegaly, patients with ventriculomegaly had a significantly higher total protein concentration in ventricular CSF (*p* = 0.047). Compared to patients without surgical complications, patients with complications had a significantly lower CSF/blood glucose ratio in ventricular CSF (*p* = 0.032). The lumbar CSF parameters had no significant differences between these groups. The changes in lumbar CSF indices over time after shunt placement were also analyzed. After shunt placement, total protein concentration was transiently increased, white blood cell count, CSF/blood glucose ratio and chloride ion concentration were continued at the preoperative level until two months after shunting surgery. These findings suggest that the composition of ventricular CSF differs from that of lumbar CSF, and different CSF parameters have disparate rostro-caudal gradients in patients with *Cryptococcal* meningitis. Furthermore, ventricular and lumbar CSF parameters may have different clinical implications. Transient deterioration of lumbar CSF parameters after ventriculoperitoneal shunt placement may not be due to disease progression, but to change in CSF flow rate by CSF shunts.

## Introduction

Cerebrospinal fluid (CSF) analysis is commonly used to identify and follow-up disease of the central nervous system (CNS)^1–3^. CSF samples are usually acquired by lumbar puncture^2,4^. In some cases, however, CSF is obtained during more invasive procedures such as external ventricular drainage (EVD) and ventriculoperitoneal shunt (VPS) placement^5–7^. Whether there is a difference between the composition of ventricular and lumbar CSF is unclear^6,8–11^. Of CSF components, the protein concentration gradient of ventricular to lumbar CSF is the most studied^8–13^. Some studies have indicated that the total protein concentration is higher in lumbar CSF than in ventricular CSF^10,11^. Most protein fractions have a higher concentration in lumbar CSF than in ventricular CSF^9,11^, while some protein fractions have the opposite concentration gradients^9,11^. Kamat et al. reported no difference of total protein level between ventricular and lumbar CSF in patients with tuberculous meningitis^8^. On the other hand, Figaji et al. reported contradictory results^14,15^. Although there are no normal reference ranges for various parameters of ventricular CSF, analysis of ventricular CSF may be used for evaluating certain diseases, such as meningeal carcinomatosis by detecting malignant cells and subarachnoid hemorrhage by detecting red blood cells. To date, there have been rare reports of ventricular CSF and CSF gradients in the study of human disease^6–12,16^.

*Cryptococcal* meningitis (CM) is a subacute, but a life-threatening fungal infection of the CNS. Non-HIV infected patients with CM are more likely to have intractable, high intracranial pressure, which is the leading cause of death and poor outcomes^17^. Hydrocephalus was also common in CM. A VPS placement is effective in treating persistent increased CSF pressure and hydrocephalus. According to previous studies on hydrocephalus, VPS placement has a high complication rate^18–23^. Hence, VPS placement is usually delayed to treat CM patients, especially when the *Cryptococcal* infection is not controlled and ventricles are not enlarged. Whether CSF parameters are associated with hydrocephalus and post-VPS complications is not clear.

The study aimed to determine whether there are differences between ventricular and lumbar CSF parameters in patients with CM and whether the differences have clinical significance.

## Methods

### Patients

**Figure 1:**
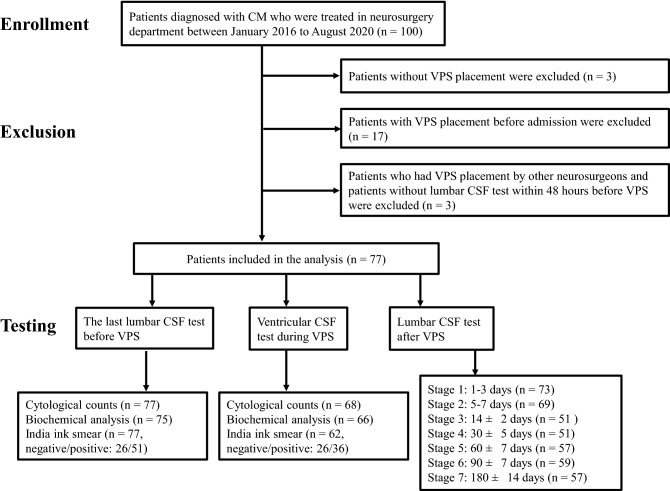
Research flow. A total of 100 patients with CM were admitted in neurosurgery department between January 2016 to August 2020. 23 patients were excluded. The number of patients who had CSF analysis at the indicated time was shown. The last lumbar CSF test before VPS was done within 48 h before surgery.

A total of 100 patients with CM were admitted to the Department of Neurosurgery of Lingnan Hospital, Branch of the Third Affiliated Hospital of Sun Yat-sen University, from January 2016 to August 2020. Only 77 patients with CM were retrospectively analyzed. The inclusion criterion was the definite diagnosis of CM. Exclusion criteria included the following: (1) Patients without VPS placement; (2) Patients who had received a shunt prior to admission to our hospital; (3) Patients who had VPS placement by other teams of neurosurgeons and patients without lumbar CSF test within 48 h before VPS (Fig. 1).

### Diagnosis of CM

A definite diagnosis of CM required at least one of the following criteria^17,24^: (1) Positive India ink staining of CSF with centrifuged sediment for *Cryptococcus*; (2) Positive culture of *Cryptococcus* from CSF; (3) Compatible histopathology (5- to 10-μm encapsulated yeast observed in brain tissue); (4) Probable CM was considered in patients with clinical symptoms of meningitis and a positive test of *Cryptococcal* antigen, and/or positive CSF findings by metagenomic next-generation sequencing. All CM patients in this study had a definite diagnosis of CM. Tuberculosis was ruled out by T-SPOT, acid-fast bacilli (AFB) smear, and ELISA.

### Treatments

Antifungal treatment before VPS placement was different based on the attending physician’s preference and the patient's tolerance of the drug. Therapeutic options are limited to amphotericin B, flucytosine, fluconazole, and voriconazole in our hospital. A relatively unified antifungal regimen of flucytosine plus fluconazole was used after VPS except for five patients (Table 1). Corticosteroids were administered to patients with immune reconstitution inflammatory syndrome. Patients with increased CSF pressure were treated with intravenous mannitol and underwent a repeated lumbar puncture before VPS placement. A VPS was considered when patients suffered from neurological deterioration caused by intractable elevated intracranial pressure and/or progressive ventricular enlargement. No patients in this study received EVD, as this option has not been usually considered at our department since 2016 due to the high frequency of secondary infection. Lumbar puncture was used to dynamically monitor CSF changes after VPS placement.

**Table 1:** Demographic and clinical features of patients.

Characteristic	No. of patients (%)	Characteristic	No. of patients (%)
**Sex**		**Pre-VPS antifungal therapy**	
Male	55 (71.4)	5-FC + Fluc	29 (37.7)
Female	22 (28.6)	AmB + 5-FC + Fluc	29 (37.7)
**Age (years)**		AmB + 5-FC	6 (7.8)
11–20	7 (9.1)	AmB + Fluc	5 (6.5)
21–40	25 (32.5)	AmB + Vori	1 (1.3)
41–60	31 (40.2)	AmB + Fluc + Vori	1 (1.3)
61–77	14 (18.2)	AmB	1 (1.3)
**Underlying conditions**		AmB + 5-FC + Fluc → AmB	2 (2.6)
Hepatitis B	12 (15.6)	AmB + 5-FC → Vori	1 (1.3)
Diabetes mellitus	3 (3.9)	AmB + 5-FC + Fluc → Fluc	1 (1.3)
Hepatitis C	2 (2.6)	Vori → AmB + 5-FC	1 (1.3)
Chronic kidney disease	2 (2.6)	**Post-VPS antifungal therapy**	
Connective tissue disease^a^	1 (1.3)	5-FC + Fluc	72 (93.5)
Malignancy^b^	1 (1.3)	AmB + 5-FC + Fluc	2 (2.6)
Normal host	52 (67.5)	Fluc	1 (1.3)
**Pre-VPS CSF pressure (mm H**_**2**_**O)**		AmB + 5-FC + Fluc → Vori	1 (1.3)
50–80	4 (5.2)	5-FC + Fluc → 5-FC + Vori	1 (1.3)
80–200	8 (10.4)	**Post-VPS complication**	
200–250	10 (13.0)	Shunt obstruction	3 (3.9)
250–330	17 (22.1)	Reinfection	1 (1.3)
330–1220	38 (49.3)	Intraventricular hemorrhage	1 (1.3)
**Pre-VPS neurological symptoms**		Abdominal pseudocyst	1 (1.3)
Headache	77 (100)	Overdrainage	1 (1.3)
Visual changes	40 (51.9)	Inadequate drainage	2 (2.6)
Hearing impairments	25 (32.4)	Postoperative epilepsy	3 (3.9)
Disturbance of consciousness	16 (20.8)	**6-month neurological outcome**	
Muscle weakness	14 (18.2)	Cured	53 (68.8)
Seizures	11 (14.3)	Better	22 (28.6)
Cognitive impairment	6 (7.8)	Worse	1 (1.3)
Urinary incontinence	2 (2.6)	Death	1 (1.3)

Data presented as n (%). AmB = amphotericin B; 5-FC = flucytosine; Fluc = fluconazole; Vori = voriconazole; NA = not applicable; + : combined with; → : changed to; ^a^systemic lupus erythematosus; ^b^multiple myeloma.

### VPS placement

The tip of the ventricular catheter was placed via a frontal approach into the foramen of Monro or the third ventricle by means of the anterior horn of the ipsilateral lateral ventricle, based on a prior definition of accurate ventricular catheter placement^25^. The non-dominant hemisphere side was the preferred side of placement. Accurate placement, which is very important to ensure all the drainage holes are in the ventricle, is essential for patients with CM without ventriculomegaly. Two types of adjustable shunt devices were used, the proGAV2.0 valve (Miethke) and the Strata II valve (Medtronic). Usually, the initial valve pressure was set to 200 mm H_2_O, or gear 2.5, respectively. The valve pressure was adjusted according to patient’s clinical response. The valve pressure would be adjusted down when a patient exhibited persistent neurological disorder, progressive ventricular enlargement, and a high opening pressure on lumbar puncture. The valve pressure would be adjusted up when a patient exhibits orthostatic headache and a low opening pressure on lumbar puncture. Prophylactic anti-epileptic drugs were usually administered peri-operatively. Shunt complications were recorded within six months after VPS. Three patients who had a shunt obstruction were successfully revised. Two patients were adjusted the depth of the tube insertion; one patient was revised by the replacement of a new shunt tube.

### Acquisition and detection of CSF parameters

We recorded the CSF testing results, which met the following requirements: Lumbar CSF samples were obtained by lumbar puncture within 48 h before VPS placement, and 1–3 days (stage 1), 5–7 days (stage 2), 14 ± 2 days (stage 3), 30 ± 5 days (stage 4), 60 ± 7 days (stage 5), 90 ± 7 days (stage 6), and 180 ± 14 days (stage 7) after VPS placement. Ventricular CSF was obtained during shunt insertion directly from the lateral ventricle (Fig. 1). CSF analysis included white blood cell (WBC) count and red blood cells (RBCs) count, total protein concentration, CSF/blood glucose ratio, chloride ion level, and *Cryptococcal* count. When visible blood contamination occurred, one leukocyte was subtracted per 1000–1500 red blood cells to estimate the true CSF WBC count^26^. *Cryptococcal* count was determined by counting the number of *Cryptococcus* per milliliter of CSF via India ink staining.

### Definition of ventriculomegaly

Ventriculomegaly was diagnosed on the basis of dilation of the temporal horn of the lateral ventricle and/or an Evans’ index of > 0.3, as determined by CT and/or MRI. Evans’ index refers to the ratio of the ventricular width of the bilateral frontal horn to the maximum biparietal diameter^22^.

### Six-month neurological outcome

All patients in the study exhibited neurological symptoms before VPS placement, such as headache, visual changes, hearing impairments, disturbance of consciousness, muscle weakness, seizures, cognitive impairment and urinary incontinence. Neurological outcomes, which were evaluated at six months after VPS placement, were divided into four levels: cured, better, worse, and death.

### Statistical analysis

All statistical analysis was performed using SPSS version 22 software (SPPS Inc., Chicago, IL, USA). All numerical variables of CSF parameters were presented as median and range or interquartile range as they were non-normally distributed data. Categorical variables were expressed as counts and percentages. A Wilcoxon signed-rank test was used to compare lumbar and ventricular CSF parameters and analyze lumbar CSF change after shunting surgery. The Mann–Whitney U test were used to analyze CSF parameters in patients with and without ventriculomegaly, and patients with and without surgical complications. Chi-squared test or Fisher’s exact test was used for comparisons of binary categorical variables, as appropriate. Statistical significance was set at a value of *p* < 0.05.

### Ethics approval and consent to participate

The Clinical Research Ethical Committee of the Third Affiliated Hospital of Sun Yat-sen University approved the study (02-167-01) and waived the requirement of written informed consent. No experiments were conducted with human participants (or their tissue). All information was kept anonymous. The study complies with the Declaration of Helsinki.

## Results

### The baseline data

A total of 77 HIV-negative patients (55 males and 22 females) with CM were included in this study, with ages ranging from 11 to 77 years. The clinical features of these patients are shown in Table 1. 67.5 percent of them were clinically normal, and others had underlying diseases as shown in Table 1. Only one patient with systemic lupus erythematosus was taking immunosuppressant before onset of CM. The majority of patients had elevated CSF pressure tested by lumbar puncture before VPS placement. One patient had a low CSF pressure of both lumbar and intraventricular CSF. Three patients had low lumbar CSF pressures (< 80 mm H_2_O) assessed by lumbar puncture, but high intraventricular CSF pressures (≥ 200 mm H_2_O) detected during shunt insertion. All of these patients received a VPS placement due to neurological deterioration caused by intractable elevated intracranial pressure and/or progressive ventricular enlargement. All of these patients exhibited headaches. Other neurological symptoms included visual changes, hearing impairment, disturbance of consciousness, muscle weakness, seizures, cognitive impairment and urinary incontinence. The antifungal therapies differed greatly before VPS surgery due to doctors’ different experiences (Table 1), but they were similar after VPS surgery since patients were treated by the same team of neurosurgeons (Table 1). 93.5 percent of patients were treated with fluconazole combined with flucytosine after VPS placement (Table 1). A total of 12 patients had post-VPS complications. All the complications were resolved with appropriate clinical management except for two patients. One patient’s drainage tube was ultimately clamped due to persistent intracranial hypotension. The other patient exhibited progressive cranial hypertension and was discharged from the hospital when brain herniation occurred one month after VPS. Six months after VPS placement, all patients’ neurological dysfunction was improved or cured except two female patients. One patient with hepatitis B virus-related decompensated cirrhosis died of hemorrhagic shock. The other patient who developed brain herniation was given up medical treatment.

### Comparison of lumbar and ventricular CSF parameters

To determine whether a rostro-caudal CSF gradient was present in patients with CM, lumbar and ventricular CSF parameters including WBC count, total protein concentration, CSF/blood glucose ratio, chloride ion concentration, and *Cryptococcal* count were compared (Table 2). The analysis revealed that all these CSF parameters were significantly different between lumbar and ventricular CSF (all, *p* < 0.001). The WBC count, total protein level, and *Cryptococcal* count were significantly lower in ventricular CSF than in lumbar CSF, while CSF/blood glucose ratio and chloride ion concentration were significantly higher in ventricular CSF than in lumbar CSF. It's important to point out that the total protein concentration in ventricular CSF of CM patients was very low with a median value of 0.12 g/L, which was different from previous reports on tuberculous meningitis^14,15^. Since there were many factors, such as manipulation, patient's position, and the bore of the puncture needle, affecting the detection of CSF pressure, the comparison of lumbar and ventricular CSF pressures was not analyzed.

**Table 2:** Comparison of lumbar and ventricular CSF parameters.

CSF parameters	Lumbar	Ventricular	Number^a^	*p* value
WBC count (cells/μL)	56.5 (2–302)	7.5 (0–111)	66	< 0.001
Total protein (g/L)	0.645 (0.20–2.81)	0.12 (0.02–1.41)	64	< 0.001
CSF/blood glucose ratio^b^	0.249 (0.002–0.677)	0.603 (0.154–0.830)	45	< 0.001
Chloride ion (mmol/L)	119.65 (104.6–229.0)	125.95 (111–143.0)	64	< 0.001
*Cryptococcal* count (/mL)^c^	998 (1–1,792,000)	48 (0–42,450)	41	< 0.001

Data presented as the median [Range].

^a^Patients who had both lumbar and ventricular CSF test.

^b^19 patients lacked contemporaneous blood glucose values.

^c^Only included patients who had positive result of India ink staining before VPS surgery.

### Comparison of CSF parameters between patients with and without ventriculomegaly before VPS

Ventricular enlargement was common in patients with CM. We analyzed lumbar and ventricular CSF parameters between patients with and without ventriculomegaly before VPS (Table 3). The age and sex distributions were not significantly different between the two groups (*p* ≥ 0.05). Interestingly, compared to patients without ventriculomegaly, patients with ventriculomegaly had higher protein concentration in ventricular CSF (*p* = 0.047). Although the levels of lumbar CSF protein of the two groups had a similar trend, they were not statistically different (*p* = 0.239). Other CSF indices in both lumbar and ventricular CSF were not significantly different between the patients with and without ventriculomegaly (all, *p* > 0.05).

**Table 3:** Comparison of CSF parameters between patients with and without ventriculomegaly before ventriculoperitoneal shunt.

Characteristic	Patients with ventriculomegaly	Patients without ventriculomegaly	*p* value
Age, year	50.0 (13–76, 19)	41.5 (11–77, 58)	0.050
Sex, M/F	13/6	42/16	0.738
**Lumbar CSF**			
WBC count (cells/μL)	60.0 (4–207, 19)	50.5 (2–568, 58)	0.679
Total protein (g/L)	0.825 (0.13–6.61, 18)	0.650 (0.20–3.75, 57)	0.239
CSF/blood glucose ratio	0.206 (0.003–0.495, 14)^a^	0.315 (0.002–0.677, 44)^b^	0.191
Chloride ion (mmol/L)	120.8 (103.0–135.1, 18)	119.2 (97.8–229.0, 57)	0.719
**Ventricular CSF**			
WBC count (cells/μL)	6.0 (0–40, 15)	8.0 (0–111, 53)	0.876
Total protein (g/L)	0.220 (0.04–0.62, 15)	0.110 (0.02–1.41, 51)	0.047
CSF/blood glucose ratio	0.515 (0.133–0.742, 12)^c^	0.630 (0.154–0.830, 42)^d^	0.088
Chloride ion (mmol/L)	127.30 (112.1–143.0, 15)	125.20 (111.0–141.9, 51)	0.201

Data presented as the median [Range, n] or n. M: male; F: female. n: data available for indicated parameters.

^a,b,c,d^4, 13, 3 and 9 patients lacked contemporaneous blood glucose values respectively.

### Comparison of CSF parameters between patients with and without post-VPS complications

High protein concentration was considered a risk factor for shunt obstruction and re-infection after VPS in hydrocephalus^21,27^. To determine whether CSF indices were associated with post-VPS complications in CM, we analyzed both lumbar and ventricular CSF parameters between patients with and without post-operative complications (Table 4). As shown in Table 1, twelve patients had surgical complications. The age and sex distributions were not statistically different between the two groups (both, *p* > 0.05) (Table 4). All lumbar CSF parameters were not significantly different between the two groups (all, *p* > 0.05). Among ventricular CSF indices, the CSF/blood glucose ratio was statistically different between the two groups (*p* = 0.032). Ventricular CSF/blood glucose ratio was lower in patients with complications than in patients without complications (Table 4).

**Table 4:** Comparison of CSF parameters between patients with and without surgical complications.

Characteristic	Patients with post-VPS complication	Patients without post-VPS complication^a^	*p* value
Age, year	48.5 (13–66, 12)	43 (11–77, 64)	0.578
Sex, M/F	10/2	45/19	0.492
**Lumbar CSF**			
WBC count (cells/μL)	73.0 (8–302, 12)	53.5 (2–568, 64)	0.397
Total protein (g/L)	0.560 (0.20–6.61, 11)	0.760 (0.13–2.81, 63)	0.903
CSF/blood glucose ratio	0.147 (0.045–0.677, 11)	0.288 (0.002–0.642, 47)^b^	0.410
Chloride ion (mmol/L)	115.30 (103.0–131.1, 11)	119.70 (97.8–229, 63)	0.254
**Ventricular CSF**			
WBC count (cells/μL)	9.5 (0–40, 10)	7.0 (0–111, 58)	0.755
Total protein (g/L)	0.175 (0.04–0.59, 10)	0.120 (0.02–1.41, 56)	0.474
CSF/blood glucose ratio	0.541 (0.154–0.625, 9)^c^	0.641 (0.133–0.830, 45)^d^	0.032
Chloride ion (mmol/L)	125.50 (112.1–143.0, 10)	125.85 (111.0–140.6, 56)	0.782

Data presented as the median [Range, n] or n.

M, male; F, female. n: Number of patients who had indicated CSF detection results.

^a^a patient who died one month after surgery due to hemorrhagic shock caused by hepatitis B virus-related decompensated cirrhosis was excluded.

^b,c,d^16, 1 and 11 patients lacked contemporaneous blood glucose values respectively.

### The effect of VPS on lumbar CSF parameters

**Figure 2:**
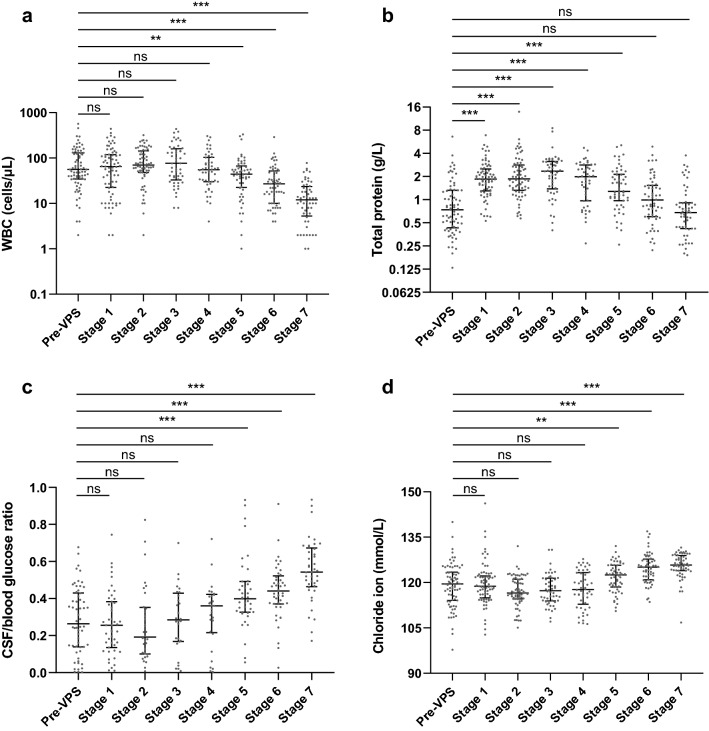
Changes of lumbar CSF parameters over time after VPS placement. (**a**) WBC count remained at the pre-operative level for at least 1 month, and then it began to decline. (**b**) Total protein level was rapidly increased after shunt placement and reached a peak at 2 weeks, then it gradually decreased. (**c**) and (**d**) CSF/blood glucose ratio and chloride ion concentration were remained at the pre-operative level during the first month after shunting surgery, and then gradually elevated. The lack of contemporaneous blood glucose values for each stage were 17, 25, 38, 22, 21, 18, 14, 15 respectively, which resulted in a low amount of CSF/blood glucose ratio data. All data are shown as median with interquartile range. ns: *p* > 0.05, **p < 0.01, ***p < 0.001 versus pre-VPS.

Since CSF diversion directly changes the CSF flow rate, which can affect the ventricular to lumbar protein concentration gradients^16^, we evaluated the changes of lumbar CSF parameters over time after VPS placement (Fig. 2a–d). The WBC count, CSF/blood glucose ratio, and chloride ion concentration remained at pre-operative levels during the first month after shunting surgery, and then they gradually returned to normal or close to normal levels. Interestingly, the total protein level rapidly increased after surgery and peaked at two weeks after VPS placement, then gradually declined to the pre-operative level at 3–6 months after surgery. Although these lumbar CSF indices seemed to be getting worse, the positive ratio of India ink staining rapidly decreased (Supplementary Fig. 1).

## Discussion

The results of this study demonstrated that the composition of ventricular CSF was different from lumbar CSF in patients with CM. Total protein level, WBC count, and *Cryptococcal* count were lower in ventricular CSF than lumbar CSF, while CSF/blood glucose ratio and chloride ion concentration were significantly higher in ventricular CSF than in lumbar CSF. The formation of concentration gradients of various components between lumbar and ventricular CSF is likely related to distinct transport mechanisms and CSF dynamics^9,28,29^. Glucose is transported through the blood–brain barrier (BBB) by facilitative and energy-dependent transport, which only occurs in the brain^29^. The chloride ion is transported by sodium–potassium-chloride cotransporters, potassium-chloride cotransporters and chloride channels in the choroid plexus^28^. Blood-derived CSF proteins diffused through the blood-CSF barrier (BCSFB) is more widely distributed in the spinal cord than in the brain^29^. The pathway of entry of leukocytes into the CSF is still unclear. We speculate that the densities of WBCs and *Cryptococcus* are diluted by newly secreted CSF in the ventricle and accumulate in the subarachnoid space of the spinal cord as CSF travels from the ventricles to the spinal channel. Sometimes *Cryptococcus* aggregates into clumps, which leads to a large *Cryptococcal* count in the CSF. Our findings were consistent with previous literature except for one report from Kamat et al. mentioned in the background section^8–12^. They found no significant differences between ventricular and lumbar protein levels in tuberculous meningitis. It seemed that the ventricular protein level in *Cryptococcal* meningitis is obviously lower than that in tuberculous meningitis. This may be the reason why hydrocephalus occurs more often in tuberculous meningitis than in *Cryptofcoccal* meningitis. Even so, we believe that there are rostro-caudal gradients from ventricular to lumbar CSF, but they vary in different diseases.

Since CM patients are more likely to have intractable high intracranial pressure and hydrocephalus that were usually treated by VPS placement, we analyzed the relationships between CSF indices and ventriculomegaly before VPS and complications after VPS. Among the CSF parameters, only ventricular CSF protein levels were statistically higher in patients with ventriculomegaly than in patients without ventriculomegaly. Our study provided further evidence that a high CSF protein level could be a risk factor for ventriculomegaly^30^. Although lumbar CSF protein level had a similar trend, it was not statistically different. It indicated that ventricular CSF protein concentration was more closely related to ventricular enlargement than lumbar CSF protein concentration. There are three hypotheses for the pathophysiological mechanism of hydrocephalus: (1) constantly elevated protein level defects reabsorption of CSF via obstruction of the arachnoid villi^31^; (2) higher protein level changes the osmotic gradient in favor of water transport into the ventricular system versus the level present at that time in blood^31,32^. (3) the abnormal CSF reflux^33,34^. Here, we propose the following hypothesis in CM: *Cryptococcal* infection induced disruption of BCSFB and BBB led to a rapid elevation of protein level in lumbar CSF and an increase of CSF secretion via choroid plexus in ventricles, which may explain why the rostro-caudal protein gradient is increased in patients with CM compared to previous study on patients with multiple sclerosis considered as an autoimmune disease^11^. The persistent high lumbar protein level would gradually increase the ventricular protein level through CSF reflux. The increased ventricular protein level may further exacerbate the CSF secretion, slow down the CSF flow and CSF reabsorption. Finally, ventriculomegaly occurred. In brief, we believe the pathophysiological changes precede the structural changes.

There was a common concern that a high CSF protein level increases the risk of shunt complications^21,35^. According to our study, there was no correlation between protein concentration and shunt complications in CM. Surprisingly, patients with post-operative complications had a significantly lower ventricular CSF/blood glucose ratio than patients without complications. This may indicate that CM patients with a lower ventricular CSF/blood glucose ratio are more likely to have shunt complications. However, which complication is the most closely correlated with CSF/blood glucose ratio requires further study. Nevertheless, the lumbar CSF/blood glucose ratio was not statistically different between the two groups. It tends to support the conjecture that ventricular CSF indices were more closely related to post-VPS complications, since shunt tubes were inserted directly into the lateral ventricles.

Since CSF diversion directly changes the CSF flow rate, which may affect the ventricular to lumbar protein gradients^16^, we evaluated the changes in lumbar CSF parameters over time after VPS placement. It seemed that WBC count, CSF/blood glucose ratio and chloride ion level had not improved until two months after VPS placement. Total protein levels were deteriorated during the first two weeks after shunt surgery. The elevated lumbar protein concentration did not return to pre-operative level until three months after shunt surgery. Based on the improvement of clinical symptoms and fast decline of CSF *Cryptococcal* count (Supplementary Fig. 1), patients benefited from VPS placement. Hence, the transient deterioration of total protein is not likely due to a worsening disease, but rather a sudden change of CSF circulation due to shunting. As mentioned previously, blood-derived CSF proteins are primarily diffused through the BCSFB in the spinal cord. The slow flow of lumbar CSF leads to an elevation of protein level, which we believe is similar to the mechanism of elevated protein levels caused by spinal canal obstruction. Likewise, WBC count, CSF/blood glucose ratio, and chloride level were also affected by shunting for at least one month until a new steady state of CSF circulation was established.

Our study is limited by its retrospective approach and a single-center clinical data. Individual subgroups were partly small, especially patients with shunt complications. Although all participants were HIV-negative CM patients, they had different comorbid conditions, which may also lead to BBB/BCSFB dysfunctions. Other influencing factors, such as antifungal therapy and detection time, also existed in this study. The values of the CSF/blood glucose ratio were missing a lot due to a lack of contemporaneous blood glucose levels. However, to our knowledge, this is the most extensive study on human ventricular CSF parameters, and this is the first study on ventricular to lumbar CSF gradient in CM.

## Conclusions

The composition of ventricular CSF differs from that of lumbar CSF in HIV-negative patients with CM. Different CSF parameters have disparate ventricular to lumbar concentration gradients. Ventricular and lumbar CSF parameters may have different clinical implications. According to our study, ventricular but not lumbar CSF indices were closely related to ventriculomegaly and shunt complications. Transient deterioration of lumbar CSF parameters after VPS placement may not be due to disease progression but rather to a change of the CSF flow rate due to CSF shunts.

## Supplementary Information

Supplementary Figure 1.

## Data Availability

The datasets used and/or analyzed during the current study are available from the corresponding author upon reasonable request.

## Abbreviations

BBB: Blood–brain barrier
BCSFB: Blood-cerebrospinal fluid barrier
CM: *Cryptococcal* meningitis
CNS: Central nervous system
CSF: Cerebrospinal fluid
HIV: Human immunodeficiency virus
RBC: Red blood cell
VPS: Ventriculoperitoneal shunt
WBC: White blood cell
