# Supplementary material for: Ventricular and lumbar cerebrospinal fluid analysis in 77 HIV-negative patients with Cryptococcal meningitis who received a ventriculoperitoneal shunt
Source: Sci Rep. 2022 Dec 9;12:21366. doi: 10.1038/s41598-022-25742-w (PMC9734559; doi:10.1038/s41598-022-25742-w)
Supplement: Supplementary file 1 — Supplementary Figure 1. [file 41598_2022_25742_MOESM1_ESM.pdf]

Title: Ventricular and lumbar cerebrospinal fluid analysis in 77 HIV-negative patients with *Cryptococcal* meningitis who received a ventriculoperitoneal shunt: a single-center retrospective study

Authors: Qing Dong [1†], Zhenchao Huang[2†], Peng Yu[2], Enpeng Song[2], Zhijie Chen[2], Feng Qin[2]*

***Correspondence:** qinfeng2@mail.sysu.edu.cn

† Qing Dong, Zhenchao Huang shared first co-first authorship.

[1]Department of Neurology, Lingnan Hospital, Branch of the Third Affiliated Hospital of Sun Yat-sen University, No.2693 Kaichuang Avenue, Guangzhou 510530, P.R. China.

[2] Department of Neurosurgery, Lingnan Hospital, Branch of the Third Affiliated Hospital of Sun Yat-sen University, No.2693 Kaichuang Avenue, Guangzhou 510530, P.R. China

Supplementary Figure

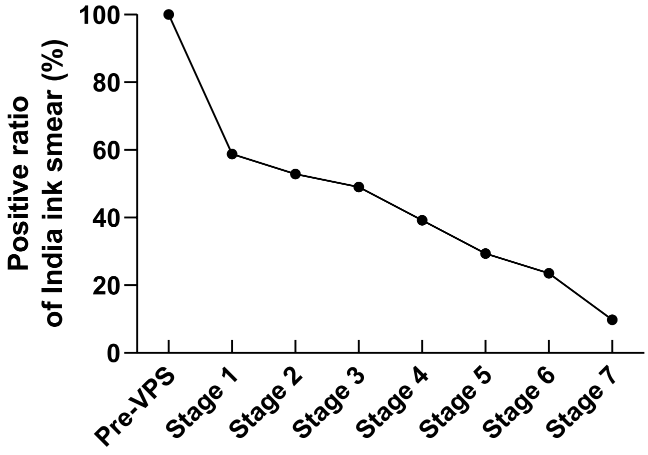

Changes of the positive ratio of lumbar CSF India ink staining after ventriculoperitoneal shunt surgery. The positive ratio was rapidly decreased at three days after surgery, then decreased slowly.
